# High‐Performance, Flexible NO_2_ Chemiresistors Achieved by Design of Imine‐Incorporated n‐Type Conjugated Polymers

**DOI:** 10.1002/advs.202200270

**Published:** 2022-03-20

**Authors:** Hyeonjung Park, Dong‐Ha Kim, Boo Soo Ma, Euichul Shin, Youngkwon Kim, Taek‐Soo Kim, Felix Sunjoo Kim, Il‐Doo Kim, Bumjoon J. Kim

**Affiliations:** ^1^ Department of Chemical and Biomolecular Engineering Korea Advanced Institute of Science and Technology (KAIST) Daejeon 34141 Republic of Korea; ^2^ Department of Materials Science and Engineering Korea Advanced Institute of Science and Technology (KAIST) Daejeon 34141 Republic of Korea; ^3^ Department of Mechanical Engineering Korea Advanced Institute of Science and Technology (KAIST) Daejeon 34141 Republic of Korea; ^4^ Department of Chemical Engineering and Materials Science Chung‐Ang University (CAU) Seoul 06974 Republic of Korea

**Keywords:** conjugated polymer‐based chemiresistors, flexible sensors, high‐performance chemiresistors, imine bonds, NO_2_ gas sensors

## Abstract

Flexible and mechanically robust gas sensors are the key technologies for wearable and implantable electronics. Herein, the authors demonstrate the high‐performance, flexible nitrogen dioxide (NO_2_) chemiresistors using a series of n‐type conjugated polymers (CPs: PNDIT2/IM‐*x*) and a polymer dopant (poly(ethyleneimine), PEI). Imine double bonds (C = N) are incorporated into the backbones of the CPs with different imine contents (*x*) to facilitate strong and selective interactions with NO_2_. The PEI provides doping stability, enhanced electrical conductivity, and flexibility. As a result, the NO_2_ sensors with PNDIT2/IM‐0.1 and PEI (1:1 by weight ratio) exhibit outstanding sensing performances, such as excellent sensitivity (Δ*R*/*R*_b_ = 240% @ 1 ppm), ultralow detection limit (0.1 ppm), high selectivity (Δ*R*/*R*_b_ < 8% @ 1 ppm of interfering analytes), and high stability, thereby outperforming other state‐of‐the‐art CP‐based chemiresistors. Furthermore, the thin film of PNDIT2/IM‐0.1 and PEI blend is stretchable and mechanically robust, providing excellent flexibility to the NO_2_ sensors. Our study contributes to the rational design of high‐performance flexible gas sensors.

## Graphical abstract

In this study, a high‐performance flexible NO_2_ chemiresistor (IM‐*x*/P‐*y*) is developed based on n‐type conjugated polymers containing imine bonds in the backbone. Excellent overall sensing performances with ultralow limit of detection (LOD) are demonstrated.

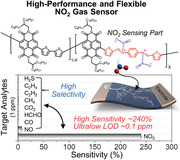

## 1 Introduction

Considering the growing demand for wearable/portable electronics, flexible gas sensors are one of the most important devices that can be integrated into health/environmental monitoring and/or implantable biomedical devices.^[^ ^1^, ^2^, ^3^, ^4^ ^]^ Conjugated polymers (CPs) are promising materials for flexible gas sensors owing to their superior optoelectrical properties, mechanical resilience, and facile solution processability compared to the conventional materials.^[^ ^5^, ^6^, ^7^ ^]^ The sensing performances (i.e., sensitivity, selectivity, reversibility, limit of detection (LOD), cycling stability, and reliability) of CPs are typically determined by the electrical modulations in the CP films during interaction with the target analytes.^[^ ^8^, ^9^ ^]^ CPs consist of alternating single and double bonds, which provide delocalized *π* electrons (*π*‐conjugations) along the backbone and allow fast charge transport. When CPs interact with gas analytes, the extent of their *π*‐conjugation can be reinforced or reduced by the partial charge transfer between the molecules and/or swelling of sensing layers, enabling transduction of chemical signals to electrical signals in the sensors. To utilize the high potential of CPs in flexible chemiresistive sensors (e.g., chemiresistors), many efforts have been made to tune the intrinsic electrochemical properties of CPs and their composites.^[^ ^10^, ^11^ ^]^

Doping is a promising strategy to amplify the sensing performance of CP‐based chemiresistors, as it can modulate the charge density and transform the base material from a semiconducting state to a highly conductive state.^[^ ^12^, ^13^ ^]^ The intensity of the electrical signal and the operation durability of the sensors are strongly influenced by the doping level and the doping stability, respectively.^[^ ^14^, ^15^ ^]^ For example, the sensing properties of p‐type polyaniline (PANI) fibers are enhanced significantly by doping with (+)‐camphor‐10‐sulfonic acid (CSA). The resistive sensor based on PANI/CSA can detect very low concentrations (1 ppm) of nitrogen dioxide (NO_2_) gas.^[^ ^16^ ^]^ Recently, an ionic liquid (IL) based on imidazolium cation and hexafluorophosphate anion has been reported for achieving the stable doping of CP sensing layers through the coupling of electronic/ionic charges.^[^ ^17^, ^18^ ^]^ The resulting CP/IL blends exhibit enhanced sensing signals, reversibility, and analyte selectivity for volatile organic compounds. Therefore, it is important to choose an appropriate dopant for the CPs, in accordance with the molecular structures of the CPs and target analytes.^[^ ^19^ ^]^

Development of new molecular structures of CPs, consisting of specific sites for selective interactions with analytes, is imperative to enhance the electrical response of the sensors. Through these interaction sites, CPs can easily donate/withdraw the electrons to/from the analyte gases and lead to large changes in electrical properties. For example, polypyrrole (PPy) was modified to include covalently linked n‐butylamine substituents, of which the amine bonds could transfer the electrons from the polymer to the target analyte.^[^ ^20^ ^]^ The modified PPy sensing layers exhibited enhanced sensitivity (e.g., 15% @ 1 ppm) with good selectivity for trinitrotoluene, whereas pristine PPy did not show any electrical response. Polythiophene (PT) block copolymers were developed to exhibit large electrical signals, where the polar polymers of second blocks could interact effectively with volatile organic compounds.^[^ ^10^ ^]^ However, these CP‐based sensors exhibit relatively poor sensitivity and LOD. This is mainly because the designated interaction sites were not highly selective to the target analytes.^[^ ^8^, ^21^, ^22^ ^]^ Therefore, it is important to develop CPs, in which the strong interaction sites for analytes are directly integrated into the conjugated backbone. In particular, monitoring NO_2_ at very low concentrations is crucial for human health or living things, because even trace levels of NO_2_ (≈1 ppm) can cause pulmonary edema and throat irritation, serving as an acute health hazard.^[^ ^11^ ^]^ Moreover, there have been only a few studies on CP‐based flexible chemiresistors.^[^ ^23^ ^]^ Therefore, the development of CP‐based NO_2_ sensors with a flexible device platform is crucial.

In this study, we develop high‐performance flexible NO_2_‐sensing chemiresistors based on imine‐incorporated n‐type CPs, PNDIT2/IM‐*x* (*x* = mole fraction of imine bond). Films consisting of PNDIT2/IM‐*x* and a polymer dopant (poly(ethyleneimine), PEI) are employed to construct the NO_2_ chemiresistors with excellent performance and mechanical flexibility. Optimal performance with a sensitivity (Δ*R*/*R*_b_) of 240% at 1 ppm of NO_2_ is obtained with the PNDIT2/IM‐0.1 film blended with PEI (1:1 by weight ratio). In this condition, the polymer‐blend film exhibits excellent selectivity against interfering gases (Δ*R*/*R*_b_ < 8% @ 1 ppm for interfering analytes). In particular, the sensor shows one of the lowest LODs (0.1 ppm) and the highest cycling stability (240% of constant sensitivity under 15 cycles of NO_2_/N_2_ exposure) among the reported CP‐based NO_2_ chemiresistors. Based on analyses using X‐ray photoelectron spectroscopy (XPS) and Raman scattering, these high sensing characteristics are mainly attributed to the strong interactions of imine bonds of the CPs with NO_2_. As the electron delocalization is disturbed by sharing the lone pair electron of $\ddot{N}$ of imine bond to NO_2_ molecules, the electrical conductivity of the sensing layer decreases rapidly. Owing to the flexible nature of the PNDIT2/IM‐*x* and PEI blends, we also demonstrate the feasibility of a flexible sensor platform using our CP blends as a sensing element.

## 2 Results and Discussion

### 2.1 Design Strategy and Characterization of System

**Figure 1:**
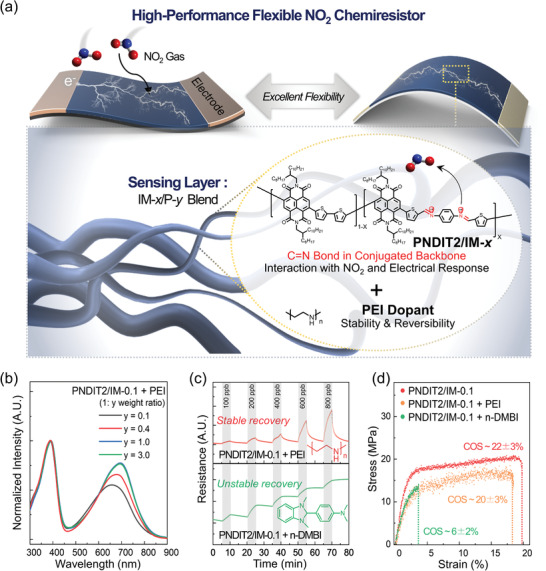
a) Schematic illustration of CP‐based sensing materials (IM‐*x*/P‐*y*) for high‐performance flexible NO_2_ chemiresistors. b) UV–vis absorption spectra of PNDIT2/IM‐*x* films blended with different weight ratios of dopant (*y*). c) Stability and recovery of IM‐*x*/P‐*y*‐based NO_2_ sensors with different dopants (i.e., PEI and n‐DMBI with the same *y* = 1.0). d) Stress–strain curves of PNDIT2/IM‐0.1 films and those with different dopants (*y* = 1.0).

To develop high‐performance flexible NO_2_‐sensing chemiresistors, we designed and synthesized a series of imine‐incorporated PNDIT2/IM*‐x* polymers by the following procedure in the previous literature^[^ ^24^ ^]^ (**Figure** **1**; Figure S1, Supporting Information). Naphthalene diimide (NDI), which is a major repeating unit of high‐performance n‐type CPs,^[^ ^25^, ^26^, ^27^, ^28^, ^29^ ^]^ was selected as one of the building blocks for the polymer backbone to generate transport pathways for the charge carriers during the sensing measurements.^[^ ^30^, ^31^, ^32^ ^]^ Another building block, (*E*,*E*)‐*N*,*N′*‐1,4‐phenylenebis[1‐(2‐thienyl)methanimine] (IM) containing C=N bonds, was prepared to achieve high‐performance sensors according to the following two considerations: (1) imine bonds (C=N) strongly interact with NO_2_ by sharing their lone pair electrons $\left(\ddot{N}\right)$; (2) these interactions affect the overall *π*‐conjugation of PNDIT2/IM*‐x*, enabling rapid changes in the electrical signals. The two different key blocks of NDI and IM were integrated to produce the PNDIT2/IM*‐x* polymers by Suzuki cross‐coupling reactions (Figure S1, Supporting Information).^[^ ^33^, ^34^ ^]^ The mole fractions of the imine bonds (*x*) in the PNDIT2/IM*‐x* polymers were varied from 0 to 0.1, and then, to 0.3. The structures of the polymers were confirmed by ^1^H nuclear magnetic resonance spectroscopy (^1^H NMR) (Figure S2, Supporting Information). The three different PNDIT2/IM‐*x* had similar, high number‐average molecular weights (*M*_n_ = 50–65 kg mol^−1^). These molecular weights of NDI polymers are higher than the critical molecular weight required to form an entanglement network in the thin film.^[^ ^35^, ^36^, ^37^ ^]^ Therefore, we expect the thin films fabricated from these polymers exhibit a high tensile strength and crack‐on‐strain (COS), which are important properties for their applications in flexible and wearable devices. To investigate the intrinsic electrical properties of the PNDIT2/IM‐*x* films and explore their feasibility in the sensors, their electron mobilities (*µ*_e_) were examined with organic field‐effect transistors (OFETs) (Figure S3, Supporting Information).^[^ ^38^ ^]^ PNDIT2/IM‐*x* exhibited a sufficiently high *µ*_e_ of 0.23, 0.27, and 0.04 cm^2^ V^−1^ s^−1^ at *x* = 0.0, 0.1, and 0.3, respectively. All the details of the methods used for the measurements and information on PNDIT2/IM‐*x* are described in the Experimental Section and Table S1 (Supporting Information), respectively.

We incorporated PEI (*M*_n_ ≈ 10 kg mol^−1^) as a polymeric dopant in the PNDIT2/IM‐*x* films to develop CP‐based chemiresistors, because of the advantages of PEI, including high n‐doping stability and mechanical ductility. Chemiresistors have a simple and effective architecture and are suitable for the flexible sensors, whereas transistor‐based sensors require additional dielectric and electrode layers, which limit the diffusion of analytes into the buried channel.^[^ ^39^, ^40^ ^]^ The doping effects of PEI on the properties of the PNDIT2/IM‐*x* thin films were examined with UV–vis absorption spectra (Figure 1b; Figure S4, Supporting Information) and electrical conductivity (Figure S5, Supporting Information). After doping, the absorption spectra of all PNDIT2/IM‐*x* exhibited blue shifts of 6–15 nm and the bandgaps reduced by 0.02–0.04 eV. Their bandgaps decreased from 1.49 to 1.43 eV when the films were doped with PEI ratios from 0.0 to 3.0 (Figure 1b). In addition, PNDIT2/IM‐0.1 showed higher electrical conductivities with larger PEI blend ratios (e.g., 1.0 × 10^−5^ and 3.1 × 10^−4^ S cm^−1^ at PEI weight ratios of 0.4 and 3.0, respectively), indicating that PNDIT2/IM‐*x* doped with PEI could provide sufficient electrical signals for chemiresistors.

Next, we investigated the feasibility of PNDIT2/IM‐*x* films doped with PEI as chemiresistor sensors, utilizing the set‐up shown in Figure S6 in the Supporting Information.^[^ ^41^ ^]^ Their sensing properties and stability were measured and compared with those of PNDIT2/IM‐*x* films doped with 4‐(1,3‐dimethyl‐2,3‐dihydro‐1*H*‐benzoimidazol‐2‐yl)phenyl)dimethylamine (n‐DMBI), which is one of the representative small‐molecule n‐doping reagents (Figure 1c).^[^ ^13^, ^42^ ^]^ The blend films (thickness ≈ 0.1–0.2 µm) consisting of dopant and PNDIT2/IM‐0.1 (1:1 by weight ratio) were prepared by drop‐casting the polymer solutions (5 mg mL^−1^ in chloroform) onto alumina substrates on which Au parallel electrodes (25 and 70 µm of width and separation distance, respectively) were deposited. The resistance of the sensor was measured and converted into a sensitivity value, i.e., ∆*R* = (*R*_gas_−*R*_b_)/*R*_b_ (%), where *R*_gas_ and *R*_b_ denote the resistance in target gas and baseline gas (N_2_), respectively. As evident in Figure 1c, the resistance increased upon exposure to 0.1–0.8 ppm of NO_2_ gas in N_2_ balance at a slightly elevated temperature (≈60 °C). Importantly, the sensor functionalized with a PEI dopant showed a good reversibility of resistance during NO_2_ sensing, which could be attributed to the high doping stability of PEI onto PNDIT2/IM‐0.1 even with NO_2_ injections. They also enabled scalable detection with a gradual increase of ∆*R* (from 2.7 to 32.6 MΩ) when the concentration of NO_2_ was increased from 0.2 to 0.8 ppm. However, the sensor with n‐DMBI showed a continuous upward drift of the resistance even after the recovery process (N_2_ injection), indicating a poor reversibility of the sensor. Similar to the most organic n‐type dopants, n‐DMBI is intrinsically unstable in the composite, owing to the high‐lying level of the highest occupied molecular orbital (HOMO) and/or low compatibility with CP chains.^[^ ^43^, ^44^ ^]^

Next, we investigated the mechanical properties of the sensing layers with different dopants through the pseudo‐free‐standing tensile test.^[^ ^45^ ^]^ This method enables the measurement of intrinsic tensile properties of thin films without any influence from the substrate. For example, the tensile strength and COS, which are highly correlated with the flexibility and ductility of the films in the sensors, can be obtained by this method.^[^ ^6^, ^46^, ^47^ ^]^ In the stress–strain curves and optical images of the tensile films (Figure 1d; Figure S7, Supporting Information, respectively), the film of PNDIT2/IM‐0.1 exhibited excellent mechanical resilience with a high COS value of 22%, which was consistent with the previous results from NDI‐based CP films with molecular weights higher than the critical molecular weight.^[^ ^37^ ^]^ Notably, the PNDIT2/IM‐0.1 film doped with PEI (1:1 by weight ratio) exhibited very similar mechanical properties with a high COS value (above 20%). This indicates that the incorporation of PEI polymer chains does not compromise the mechanical properties of the film, which is important for their use in flexible sensors. In contrast, the film with n‐DMBI showed cracks at a very low external strain (≈6%) because the n‐DMBI molecules in thin films served as the initial crack point and led to high roughness of the films. Therefore, the blend films of PNDIT2/IM‐*x* with PEI polymer dopant are employed for constructing NO_2_ sensors in this study.

### 2.2 Gas Sensing Properties of CP‐Based Chemiresistors

**Figure 2:**
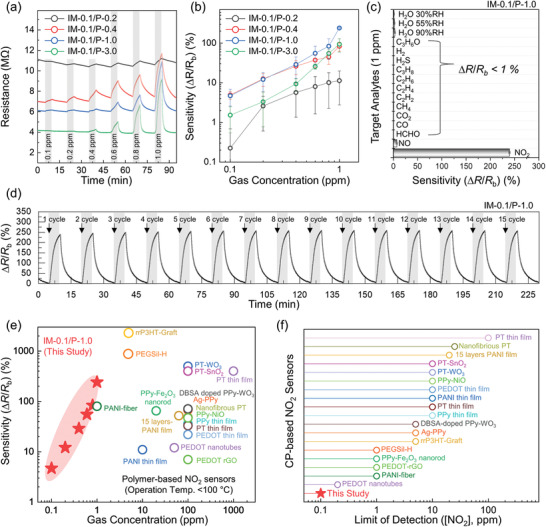
a) Dynamic resistance variations and b) gas sensitivity upon exposure to 0.1–1 ppm NO_2_ gas using IM‐0.1/P‐*y*‐based sensors with different weight ratios of PEI dopants. c) Selective gas sensing properties of IM‐0.1/P‐1.0 toward NO_2_, NO, HCHO, CO, CO_2_, CH_4_, C_2_H_2_, C_2_H_4_, C_2_H_6_, C_3_H_8_, H_2_S, H_2_, C_3_H_6_O, and H_2_O (30%, 55%, and 90% RH) gas analytes. d) Long‐term stability of IM‐0.1/P‐1.0 sensors toward cyclic exposure to 1 ppm NO_2_ gas using IM‐0.1/P‐1.0 sensors. Comparison of the e) NO_2_ sensitivity and f) LOD of IM‐0.1/P‐1.0 sensors with those of previously reported CP‐based sensors.

To obtain the optimal sensitivity of PNDIT2/IM‐*x* with PEI dopants, a series of PNDIT2/IM‐*x* films with different weight ratios of PEI (IM‐*x*/P‐*y*) were compared, where *y* is the weight ratio between PEI and PNDIT2/IM‐*x* in the blend film. We systematically investigated the sensing characteristics of the IM‐*x*/P‐*y*‐based sensors at different operating conditions (e.g., operating temperature, gas concentration, etc.). First, the effect of the operating temperature was examined using the IM‐0.1/P‐1.0 sensor by varying the temperature from room temperature (RT ≈ 20 °C) to 90 °C (Figure S8, Supporting Information). Not only the reversibility but also the sensitivity (∆*R*/*R*_b_ ≈ 240% @ 1 ppm NO_2_) increased significantly at an elevated temperature (60 °C), while a very high operation temperature (90 °C) led to a large decrease in the sensitivity (∆*R*/*R*_b_ ≈ 20% @ 1 ppm NO_2_). According to the Schaefer–Siebert–Roth model, the use of high temperatures can promote the hopping of charge carriers through CPs, resulting in enhanced conductivity and sensor performance.^[^ ^48^ ^]^ However, the dedoping process can be accelerated at elevated temperatures, which decreases the sensitivity.^[^ ^12^ ^]^ Thereafter, we varied the PEI doping content (*y*) from 0.2 to 3.0 in the blend with IM‐0.1 to observe the effect on sensitivity (**Figure** **2**a). The baseline resistance of the IM‐*x*/P‐*y*‐based sensors decreased from 11.1 to 7.0, 6.1, and 4.2 MΩ as the *y* value increased from 0.2 to 0.4, 1.0, and 3.0, respectively. Based on the sensitivity graph at each gas concentration (0.1–1 ppm), we observed that the IM‐0.1/P‐1.0 sensor exhibited the highest sensitivity (∆*R*/*R*_b_ = 4.7% @ 0.1 ppm and 240.4% @ 1 ppm) toward NO_2_ (Figure 2b). These sensitivities were 20.9‐ and 18.3‐fold enhanced at 0.1 and 1 ppm, respectively, compared to that of the IM‐0.1/P‐0.2 sensor. Moreover, the IM‐0.1/P‐1.0 sensor exhibited high sensitivity with the detection of an extremely low concentration of 0.1 ppm NO_2_. We further investigated the effect of imine content (*x*) on the electrical resistance of the blend films and gas sensing properties (Figure S9, Supporting Information). As *x* increased from 0.0 to 0.1 and to 0.3, the baseline resistance changed from 6.3 to 4.6 and to 12.5 MΩ, respectively, following a similar trend of the electron mobility of the pristine PNDIT2/IM‐*x* films depending on the *x* values. In terms of the sensing characteristics, the sensor with IM‐0.3/P‐1.0 showed a reduced sensitivity (e.g., 33.5% degradation at 0.1–1 ppm NO_2_) compared to the sensor with IM‐0.1/P‐1.0, indicating that 0.1 is the optimal mole fraction of the imine bonds for the NO_2_ sensors.

Next, we investigated the selectivity and humidity/cycling stability of the sensors (Figure 2; Figures S10 and S11 and Table S2, Supporting Information). We selected the IM‐0.1/P‐1.0‐based sensor as it showed optimal sensitivity. The selective gas‐sensing characteristics of the IM‐0.1/P‐1.0 sensor were examined upon exposure to 1 ppm of the representative environmentally hazardous gas analytes including NO_2_, nitric oxide (NO), formaldehyde (HCHO), carbon monoxide (CO), carbon dioxide (CO_2_), methane (CH_4_), acetylene (C_2_H_2_), ethylene (C_2_H_4_), ethane (C_2_H_6_), propane (C_3_H_8_), hydrogen sulfide (H_2_S), hydrogen (H_2_), acetone (C_3_H_6_O), and water vapor (H_2_O) with 30%, 55%, and 90% relative humidity (RH) conditions (Figure 2c; Figure S10, Supporting Information). A high cross‐selectivity value (*S*_NO2_/*S*_inter_ > 32.2, where *S*_NO2_ and *S*_inter_ denoted the sensitivity toward NO_2_ and interfering gas analytes, respectively) was obtained, which is superior to those of the previously reported CP‐based gas sensors.^[^ ^49^, ^50^, ^51^ ^]^

Long‐term stability is a major challenge for CP‐based gas sensors, as they often experience an incomplete recovery of conductivity after chemical reactions with target analytes. To investigate the cycling stability of the IM‐0.1/P‐1.0 sensor, cycling gas exposure tests, i.e., 1 ppm NO_2_ for 15 cycles of NO_2_/N_2_ injections were conducted (Figure 2d). During the cycling tests, consistent and reversible resistance variations were obtained without a noticeable degradation of sensitivity (∆*R*/*R*_b_ = 240.4 (±1.6) % @ 1 ppm). For real‐world applications of CP‐based gas sensors, the sensing characteristics should be measured in air and under various humid conditions. Therefore, we investigated the sensing behaviors of the IM‐0.1/P‐1.0 sensor under different conditions of RH, including 1.5% (dry air), 55%, and 90% in air atmosphere (Figure S11, Supporting Information). Stable baseline resistance and reversible resistance variations of the IM‐*x*/P‐*y*‐based sensors were observed, indicating their promising feasibility under different RH conditions. When comparing the sensing performance of IM‐*x*/P‐*y* with that of other previous CP‐based NO_2_ chemiresistors (Figure 2e,f), the IM‐0.1/P‐1.0‐based sensors show one‐of‐the highest sensitivity at low NO_2_ concentrations. In addition, they exhibit the lowest LOD (0.1 ppm), which is superior to those of any other CP‐based sensors (**Table** **1**; Table S2, Supporting Information).

**Table 1:** State‐of‐the‐art CP‐based NO_2_ sensors operating at mild temperature (<90 °C). Types of CPs and their abbreviations: polypyrrole (= PPy), polythiophene (= PT), poly(phenyl vinylene) (= PPV), polyaniline (= PANI), polyacetylene (= PA), polyethylene glycol (= PEG), poly(3‐hexyltiophene) (= P3HT), polymethylsiloxane (= PMS)

Sensing element	Sensing temperature [°C]	Sensitivity [%]	LOD [ppm]	Cyclability [# of cycles]	Recovery Time [S]	Ref
PPy thin film	RT	36 @ 100 ppm	10	3	2170 @ 100 ppm	[49]
15‐layers PANI film	RT	65 @ 20 ppm	20	nr	nr	[64]
PT film	60	400 @ 1000 ppm	100	nr	nr	[65]
PT thin film	RT	33 @ 100 ppm	10	3	220 @ 100 ppm	[66]
Interconnected nanofibrous PT thin film	RT	48 @ 100 ppm	25	4	80 @ 100 ppm	[67]
PEDOT nanotubes	RT	52 @ 63 ppm	0.2	2	nr	[51]
PANI thin film	RT	11 @ 10 ppm	10	nr	420 @ 100 ppm	[68]
Ag‐PPy	RT	68 @ 100 ppm	5	nr	1777 @ 50 ppm	[69]
PANI‐fibers	RT	80 @ 1 ppm	1	6	63 @ 500 ppm	[16]
PEDOT film	80	22 @ 100 ppm	10	nr	nr	[50]
PEDOT‐rGO	80	7 @ 100 ppm	1	5	nr	[70]
DBSA‐doped PPy‐WO_3_	RT	71 @ 100 ppm	5	3	5990 @ 100 ppm	[71]
PPy‐NiO	RT	47 @ 100 ppm	10	4	1500 @ 60 ppm	[72]
PPy‐Fe_2_O_3_ nanorod	RT	12 @ 50 ppm	1	5	nr	[73]
PT‐WO_3_	90	500 @ 100 ppm	10	nr	nr	[74]
PT‐SnO_2_	90	400 @ 100 ppm	10	nr	nr	[75]
rrP3HT‐PMS Graft	50	2300 @ 5 ppm	5	3	nr	[76]
PEGSil‐H	50	900 @ 5 ppm	1	Nr	nr	[77]
IM‐0.1/P‐1.0	60	240 @ 1 ppm	0.1	15	228 @ 1 ppm	This Study

### 2.3 NO_2_ Sensing Mechanism

**Figure 3:**
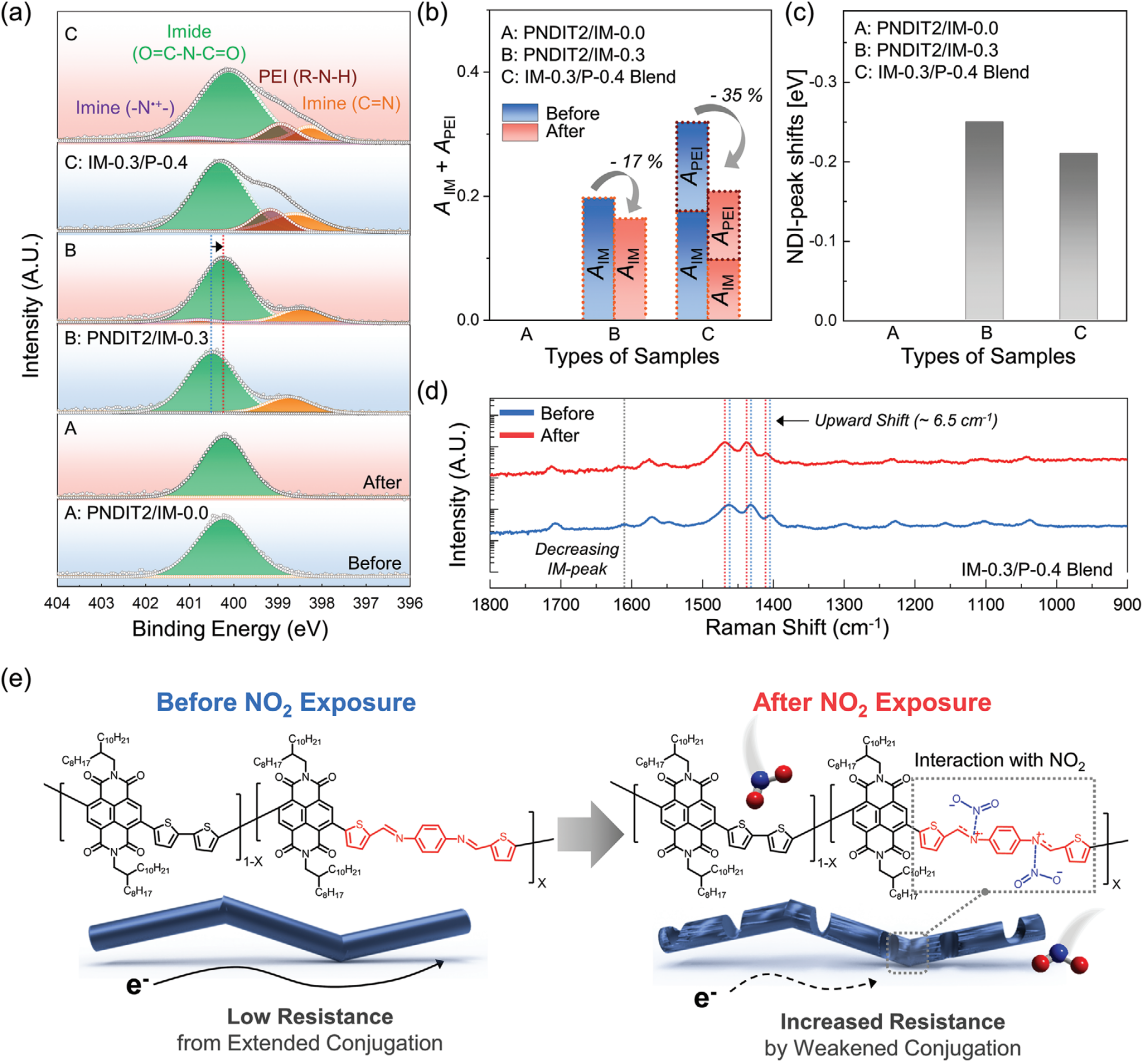
a) XPS analysis of N 1s spectra of A) PNDIT2/IM‐0.0, B) PNDIT2/IM‐0.3, and C) IM‐0.3/P‐0.4 blend films before (blue background) and after (red background) NO_2_ exposure. b) Changes in the relative peak areas (*A*_IM_ + *A*_PEI_) before and after NO_2_ exposure. c) NDI‐peak shift before and after NO_2_ exposure. d) Raman spectra of IM‐0.3/P‐0.4 blend films before and after NO_2_ exposure. e) Illustration of the suggested NO_2_ gas sensing mechanism.

The sensing mechanism of the IM‐*x*/P‐*y‐*based NO_2_ sensor was investigated through XPS measurements of the sensing layers before and after NO_2_ exposure (**Figure** **3**).^[^ ^52^ ^]^ To clarify the effect of each component during NO_2_ sensing, pristine PNDIT2/IM‐0.0, pristine PNDIT2/IM‐0.3, and IM‐0.3/P‐0.4 blend films were compared in terms of their chemiresistive sensing measurements (Figure 3a). All the films were prepared by the same methods of sensor measurements. The XPS data of three different samples were mainly discussed through the N 1s spectra, as no distinct changes were observed in O 1s and C 1s spectra. First, PNDIT2/IM‐0.0 exhibited a single imide (O=C–N–C=O) peak from the NDI moiety at 400.2 eV (NDI‐peak), which is well‐matched with the results of the previous studies.^[^ ^53^, ^54^, ^55^ ^]^ Meanwhile, after the integration of IM into NDI, PNDIT2/IM‐0.3 showed an additional peak at 398.5 eV, associated with the C=N bonds of IM (IM‐peak).^[^ ^53^, ^56^, ^57^ ^]^ Afterward, when the PEI dopant was introduced, the IM‐0.3/P‐0.4 blend sample exhibited an enhanced intensity of the peak between 397 and 400 eV, where the IM‐peak and PEI‐peak (from the R–N–H bond of PEI) overlapped.^[^ ^53^, ^55^ ^]^

Next, we investigated the changes of the area and position of each peak before and after NO_2_ exposure. To quantify the interactions of each component with NO_2_ analytes, the strengths of the IM‐ and PEI‐peaks were estimated in terms of the relative ratio of peak areas (*A*_IM_ and *A*_PEI_, respectively), which is the integrated peak areas of IM‐ and PEI‐peaks divided by the summation of the areas of all the peaks, respectively (Figure 3b). At the same time, because there was a whole shift of spectra, NDI‐peak was used to determine the modulated doping levels of the conjugated backbone. This is estimated in terms of NDI‐peak shift, which is the difference of binding energy of NDI‐peak before and after NO_2_ exposure (Figure 3c). In PNDIT2/IM‐0.0, there was no change in the area or position of NDI‐peak after NO_2_ exposure, suggesting that the NDI unit did not interact with NO_2_. In contrast, PNDIT2/IM‐0.3 exhibited a slight decrease in the IM‐peak intensity after NO_2_ exposure (≈17% decrease in *A*_IM_) and also showed an additional peak at 400.8 eV. These changes suggest that an interacted state of imine bond with NO_2_ (–N^•+^–) is formed and the imine bonds might be weakened via NO_2_ interactions. We note that the molecular structures of PNDIT2/IM‐*x* can be preserved even after long‐term exposure of NO_2_, despite the strong interaction between C=N and NO_2_, as evidenced by no noticeable change in the ^1^H NMR and UV–vis absorption spectra of PNDIT2/IM‐*x* films before and after continuous NO_2_ exposure for 3 d (Figure S12, Supporting Information). Furthermore, the IM‐0.3/P‐0.4 blend exhibited the same additional peak of –N^•+^– and larger decreases in *A*_IM_ + *A*_PEI_ (≈35%) than pristine PNDIT2/IM‐0.3. The decreases could be attributed to not only the weakened imine bonds (*A*_IM_), but also the dedoping of PEI (*A*_PEI_) by NO_2_ interactions (Figure 3b). Supported by the XPS analysis of PNDIT2/IM‐0.0 with PEI (i.e., IM‐0.0/P‐0.4), the influence of PEI dedoping might be the same in IM‐*x*/P‐*y*‐based sensors with different imine contents (*x*), because the decreasing ratios of *A*_PEI_ were very similar in both imine contents (decreasing ratio of *A*_PEI_ = 17% and 18% at *x* = 0.0 and 0.3, respectively, in Figure S13, Supporting Information). For both the PNDIT2/IM‐0.3 and IM‐0.3/P‐0.4 blend samples, NDI‐peak shifted toward a lower binding energy (≈0.2–0.3 eV) because of the electron localization by the weakened conjugations (Figure 3c). In short, the adsorption of NO_2_ onto PNDIT2/IM‐*x* was driven by two mechanisms. First of all, C=N bond of PNDIT2/IM‐*x* donates its lone pair electrons $\left(\ddot{N}\right)$ to NO_2_ molecules. This causes a larger electron‐deficiency of the C=N bond, which disturbs the push‐pull structures of PNDIT2/IM‐*x* and weakens the backbone conjugation.^[^ ^58^ ^]^ Second, dedoping of PEI from the PNDIT2/IM‐*x* composites reduces the charge‐carrier density of CPs.^[^ ^53^, ^55^ ^]^ These changes resulted in a rapid increase in the electrical resistance, enabling the detection of very low concentrations of NO_2_.

The molecular interactions between NO_2_ and the IM‐*x*/P‐*y* films were also supported by Raman spectra (Figure 3d). Before NO_2_ exposure, IM‐0.3/P‐0.4 represented similar spectra with those of conventional NDI‐based CPs, reported in the previous literature.^[^ ^59^ ^]^ An additional peak at ≈1574 cm^–1^ was observed from the intensity of C=N stretching vibrations.^[^ ^60^ ^]^ After NO_2_ exposure, the band from C=N stretching was decreased and the overall Raman spectra exhibited upward shifts (≈6.5 cm^−1^). The upward shift of Raman spectra was highly correlated with the reduction of electron delocalization in the CP chains.^[^ ^61^, ^62^ ^]^ Considering all these observations, the NO_2_ sensing mechanism of the IM‐*x*/P‐*y*‐based sensor is proposed (Figure 3e). Before NO_2_ exposure, a high charge‐carrier density in the active channel by PEI doping and extended conjugations of CPs chains successfully lead to a high electrical conductivity and low baseline resistance of sensors. After NO_2_ exposure, the CP chains are influenced by two effects: (1) the weakening of the conjugations by donating the lone pair electrons of C=N bonds to NO_2_ analytes; and (2) the reduction of the charge‐carrier density in CPs by the dedoping of PEI. Accordingly, rapid increases in resistance enable the sensors to detect very low concentrations of NO_2_ gas with high sensitivity, long‐term stability, and high selectivity.

### 2.4 Applications in Flexible and High‐Performance NO_2_ Sensor

**Figure 4:**
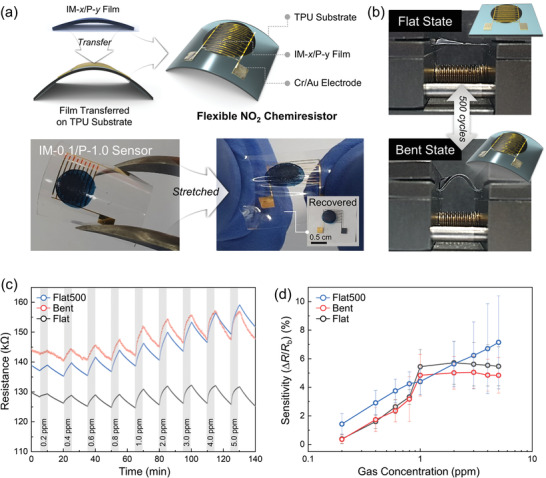
a) Illustrations of fabrication of the flexible IM‐*x*/P‐*y* chemiresistor on the electrode‐deposited TPU substrate. Photographic images show the stretching and releasing of the sensor. b) Photographic images of the sensor in the flat and bent states under cyclic bending deformations. c) Dynamic *R*_b_ and d) corresponding sensitivity values of the IM‐0.1/P‐1.0‐based sensors under Flat, Bent, and Flat500 conditions upon exposure to 0.2–5 ppm of NO_2_ gas.

To demonstrate the feasibility of IM‐*x*/P‐*y* in flexible sensor applications, we fabricated a flexible and stretchable device as follows. We used a mechanically stable thermoplastic polyurethane (TPU) substrate^[^ ^63^ ^]^ (thickness ≈ 300 µm) with Cu/Au bilayer electrodes (5 nm/100 nm) (**Figure** **4**a). The polymer solutions of IM‐0.1/P‐1.0 were drop‐casted on the prepared poly(sodium 4‐styrenesulfonate) (PSS)‐coated glass; Thereafter, the polymer films were floated on water by dissolving the PSS sacrificial layer and transferred onto the TPU substrates. Next, we measured the performance of the flexible sensors under three different measurement conditions; (i) flat state (Flat), (ii) bent state (Bent), and (iii) flat state after 500 cycles of bending tests (Flat500) (Figure 4b). The bending tests were conducted using a homemade bending machine with a bending angle of ≈80 ° and a bending radius of 1.9 mm. The NO_2_ sensing properties were analyzed upon exposure to 0.2–5 ppm of NO_2_ in N_2_ atmosphere at RT. All the sensors showed similar baseline resistances (130, 144, and 138 kΩ at Flat, Bent, and Flat500, respectively) and reliable reversibility (Figure 4c,d). As the IM‐*x*/P‐y based sensing material features excellent flexibility and mechanical stability, the sensor with IM‐0.1/P‐1.0 could monitor very low concentration (0.2 ppm) of NO_2_ analytes in both Bent and Flat500 states at RT.

## 3 Conclusion

In this study, we demonstrated high‐performance flexible chemiresistive NO_2_ sensors with a newly designed CP‐based system (IM‐*x*/P‐*y*). The incorporation of imine bonds into the conjugated backbone could facilitate good interaction with NO_2_, while all‐polymer‐based sensing materials provided excellent mechanical stability. The sensing properties were investigated systemically under different operating conditions, such as different sensing temperatures, mole fractions of imine bonds, weight ratios of PEI dopants, RH in atmosphere, and cyclic exposure of gas. IM‐0.1/P‐0.1 exhibited outstanding performances in terms of high sensitivity (Δ*R*/*R*_b_ = 240% @ 1 ppm of NO_2_), selectivity (*S*_NO2_/*S*_inter_ > 32.2), ultralow LOD (≈0.1 ppm), and high long‐cycling stability. Based on XPS and Raman spectroscopic analyses, the unique sensing mechanisms were identified: the effective modulations of the conjugations in IM‐*x*/P‐*y* were derived by donating the lone pair electrons of the imine bonds to NO_2_ analytes and dedoping of PEI dopant, leading to large modulations in the electrical signals. Furthermore, we successfully demonstrated the substantial potential of IM‐*x*/P‐*y* as a flexible sensor platform. The resulting sensor in the bent state or after subjecting to 500 bending cycles could monitor sub‐ppm level of NO_2_ gas with high sensitivity.

## Conflict of Interest

The authors declare no conflict of interest.

## Supporting information

Supporting Information

## Data Availability

The data that support the findings of this study are available from the corresponding author upon reasonable request.
