# Supplementary material for: High‐Performance, Flexible NO2 Chemiresistors Achieved by Design of Imine‐Incorporated n‐Type Conjugated Polymers
Source: Adv Sci (Weinh). 2022 Mar 20;9(14):2200270. doi: 10.1002/advs.202200270 (PMC9109064; doi:10.1002/advs.202200270)
Supplement: Supplementary file 1 — Supporting Information [file ADVS-9-2200270-s001.pdf]

ADVANCED SCIENCE
Open Access

Supporting Information

for *Adv. Sci.*, DOI 10.1002/advs.202200270

High-Performance, Flexible NO_2 Chemiresistors Achieved by Design of Imine-Incorporated n-Type Conjugated Polymers

Hyeonjung Park, Dong-Ha Kim, Boo Soo Ma, Euichul Shin, Youngkwon Kim, Taek-Soo Kim, Felix Sunjoo Kim, Il-Doo Kim and Bumjoon J. Kim**

© 2022 Wiley-VCH GmbH

Supporting Information

High-Performance, Flexible NO_2 Chemiresistors Achieved by Design of Imine-Incorporated n-Type Conjugated Polymers

Hyeonjung Park[a,†]*, Dong-Ha Kim*[b,†]*, Boo Soo Ma*[c]*, Euichul Shin*[b]*, Youngkwon Kim*[a]*, Taek-Soo Kim*[c]*, Felix Sunjoo Kim*[d]*, Il-Doo Kim*[b,]***, and Bumjoon J. Kim*[a,]**

[a]H. Park, Y. Kim, B. J. Kim
Department of Chemical and Biomolecular Engineering
Korea Advanced Institute of Science and Technology (KAIST)
Daejeon 34141, Republic of Korea
E-mail: bumjoonkim@kaist.ac.kr

[b]D.-H Kim, E. Shin, I.-D. Kim
Department of Materials Science and Engineering
Korea Advanced Institute of Science and Technology (KAIST)
Daejeon 34141, Republic of Korea
E-mail: idkim@kaist.ac.kr

[c]B. S. Ma, T.-S. Kim
Department of Mechanical Engineering
Korea Advanced Institute of Science and Technology (KAIST)
Daejeon 34141, Republic of Korea

[d]F. S. Kim
Department of Chemical Engineering and Materials Science
Chung-Ang University (CAU)
Seoul 06974, Republic of Korea

Table of Contents

Experimental Section

Supporting Figures

- **Figure S1.** Schematic illustration of the synthesis of a) bisBpin-IM and b) PNDIT2/IM-*x*.
- **Figure S2.** ^{1}H NMR spectra of a) bisBpin-IM and b) PNDIT2/IM-*x*. c) Magnified ^{1}H NMR spectra of PNDIT2/IM-*x* to determine the *x* values.
- **Figure S3.** a) Transfer curves of OFETs fabricated with PNDIT2/IM-*x*. Output curves of OFETs fabricated with b) PNDIT2/IM-0.0 c) PNDIT2/IM-0.1, and d) PNDIT2/IM-0.3.
- **Figure S4.** UV-vis absorption spectra of PNDIT2/IM-*x* films before and after doping.
- **Figure S5.** I-V curves of PNDIT2/IM-*x* films blending with different weight ratios of PEI.
- **Figure S6.** System for the gas sensor measurements.
- **Figure S7.** Optical microscopic images of PNDIT2/IM-*x* films under tensile test.
- **Figure S8.** a) Dynamic resistance variations and b) sensitivity of IM-0.1/P-1.0 at controlled operating temperatures.
- **Figure S9.** a) Dynamic resistance variations and b) sensitivity of IM-*x*/P-*y* with different IM contents.
- **Figure S10.** Humidity sensing properties of IM-0.1/P-1.0-based sensors upon exposure to 30, 55, and 90% RH in N_2 atmosphere.
- **Figure S11.** a) Dynamic resistance variations and b) sensitivity of IM-0.1/P-1.0 at controlled humidity conditions.
- **Figure S12.** a) ^{1}H NMR spectra and b) UV-vis absorption spectra of PNDIT2/IM-0.1 before and after exposure NO_2 (1 ppm) for 3 days.
- **Figure S13.** XPS analysis of N 1s spectra of the films of PNDIT2/IM-0.0 with PEI before (blue background) and after (red background) NO_2 exposure.

Supporting Tables

- **Table S1.** Characteristics of PNDIT2/IM-*x* used in this study
- **Table S2.** Gas sensing properties of IM-0.1/P-*y*

Experimental Section

Materials

General reagents, commercially purchased from Sigma Aldrich, were used for the chemical reaction without an additional purification process. The anhydrous solvents were used for chemical reactions. The column chromatography carried out on Merck silica gel 60 was applied for purifications of all the products and eluents of the column were industrial grade. All the reagent and catalysts for Suzuki cross-coupling reactions and 10 kg mol^{-1} of linear-PEI were purchased from Sunatech Inc. and Tokyo Chemical Industry.

Synthesis of PNDIT2/IM-x

5-Bromo-2-carbaldehyde-thiophene was produced from 2-carbaldehyde-thiophene (10 g, 89 mmol) reacted with the *N*-bromosuccinimide (NBS, 15.8 g, 89 mmol) dissolved in dry tetrahydrofuran (THF, ~100 mL). The reacted solution was stirred for 24 h under a nitrogen (N_2) atmosphere at 0 °C in a dark condition, followed by quenching and purifications with silica gel chromatography (hexane). Then, 10 g of yellow oil product (5-bromo-2-carbaldehyde-thiophene, 52 mmol) was stirred with bis(pinacolato)diboron (15.8 g, 62.4 mmol), $PdCl_2$(dppf) (4.5 g, 5.2 mmol), and KOAc (15.3 g, 156 mmol) in anhydrous 1,4-dioxane (200 ml) at 80 °C for 6 h under N_2. As the results, 5-(4,4,5,5-tetramethyl-1,3,2-dioxaborolan-2-yl)thiophene-2-carbaldehyde was obtained after further extraction and silica gel chromatography (eluents: hexane/DCM) purification. At last, a solution of previous products (4.76 g, 20 mmol) was reacted with *p*-phenylenediamine (1.08 g, 10 mmol), *p*-toluenesulfonic acid (PTSA) (172 mg, 1 mmol), anhydrous $CaCl_2$ (10 g), and anhydrous toluene (200 mL) under vigorous stirring for 48 h at 90 °C in N_2. The final compound, *bisBpin-IM*, was achieved as a yellow solid through recrystallization using isopropyl alcohol/diethyl ether mixtures. ^{1}H NMR (400 MHz, $CDCl_3$) δ 8.62 (s, 2H), 7.62 (d, 2H), 7.55 (d, 2H), 7.27 (s, 4H), 1.36 (s, 24H).

All the NDIT2/IM-*x* were obtained by following Suzuki-coupling polymerization. The 4,9-dibromo-2,7-bis(octyldodecyl)benzo[lmn][3,8]-phenanthroline-1,3,6,8-tetraone (200 mg, 0.2 mmol) was polymerized with mixture of bithiophene monomers, which is composed with α mol of *bisBpin-IM* and β mol 2,5-bis(4,4,5,5-tetramethyl-1,3,2-dioxaborolan-2-yl)thiophene ($x = \alpha / (\alpha + \beta)$, $\alpha + \beta$ = 0.2 mmol). CsF (122 mg, 0.8 mmol) and $Pd(OAc)_2$ (1.3 mg, 6 μmol) were put in a microwave vessel (5 mL) as catalysts to efficiently achieve high molecular weights of NDIT2/IM-*x*. Then, the compounds were vigorously stirred at 110 °C for 12 h

under argon atmosphere after the addition of 4 mL of anhydrous toluene and 0.4 mL of *N*,*N*-dimethyl methanamide (DMF). To purify the polymers, mixtures were precipitated in cold methanol (100 mL) and washed by soxhlet extraction with methanol, acetone, and hexane. Final products were obtained as dark powders by precipitation of chloroform-soluble fraction.

Characterizations of PNDIT2/IM-x and blends with PEI (IM-x/P-y)

The size exclusion chromatography (SEC), of which a standard and an eluent were polystyrene and dichlorobenzene (*o*-DCB), respectively, was used for measuring the molecular weights of the PNDIT2/IM-*x*. The SEC was conducted with a flow rate of 1 mL min^{-1} at 80 °C. The Waters 1515 and Waters 2414 were used for an isocratic pump operating and a refractive index detector, respectively. The chemical structures of all reactants and PNDIT2/IM-*x* were characterized by ^{1}H NMR spectra measured by Bruker Avance 400 MHz spectrometers at a high temperature (~ 110 °C and the samples were prepared in deuterated *o*-DCB (δ 7.17 and 7.40 ppm) (Figure S2).

The absorption spectra of thin films of IM-*x*/P-*y* were obtained by the Bruker Optics and Shimadzu Model UV-1800 spectrometers. The samples were prepared by spin-coating of blend solutions (in CF with 10 mg mL^{-1}) onto the washed glasses. Their electrical conductivities (σ) were measured by the four-point probe method, using a Keithley 2400 source meter. The conductivity parameter can be calculated from the current-voltage (I-V) curves by using the following equation:

$$\sigma = \frac{1}{k}\frac{I}{V}$$

where k is the correction factor including the geometric parameter of measurements.

OFETs

PNDIT2/IM-*x* films were prepared by spin-coating of polymer solutions (in CF with 10 mg mL^{-1}) onto the *n*-octadecyltrichlorosilane treated SiO_2/Si wafers (dielectric layers, thermally grown SiO_2 ~200 nm, C_i = 17.3 nF cm^{-2}) and then annealing at 220 °C for 20 min under N_2. The gold (Au) electrode (~ 40 nm of thickness) was deposited on the polymer/dielectric films through a shadow mask with 1000 μm of width (W) and 40 μm of length (L). Finally, prepared OFETs were bottom-gate top-contact configurations of gate structures. The transfer curves were obtained by a Keithley 4200 semiconductor parameter analyzer under N_2. The OFET parameters can be calculated from the transfer curves by using the following metal-

oxide-semiconductor field-effect transistor equation:

$$I_D = \frac{1}{2}\frac{W}{L}\mu_e C_i (V_{\mathrm{G}} - V_{\mathrm{Th}})^2$$

where I_{D} is the drain current, V_{G} is the gate voltage, V_{Th} is the threshold voltage, and C_{i} is the areal capacitance of the dielectric layer.

Pseudo-Free-Standing Tensile Test

The pseudo-freestanding tensile test was conducted by following procedures. The polymer films (~150 nm of thickness) were prepared by spin-casting of PNDIT2/IM-*x* solution (in CF with 10 mg mL^{-1}) onto poly(styrenesulfonate) (PSS)-coated glasses. Then, the layered samples with polymer film/PSS/glass were cut into bar-type specimens (5 mm × 25 mm sizes). For pseudo-freestanding tensile tests, the PNDIT2/IM-*x* films were floated onto water, while dissolving the PSS layers and remaining only the polymer layers. After the specimen was contacted with the polydimethylsiloxane-coated aluminum (Al) grips, the tensile test was proceeded at 25 °C using a linear stage with a strain rate of 2 μm s^{-1}. The stress and strain were obtained by a load cell (LTS-10GA, KYOWA) and a digital image correlation device, respectively.

Chemiresistors on Alumina Substrate or Flexible TPU substrate

The polymer solutions of IM-*x*/P-*y* were prepared by the mixture of PNDIT2/IM-*x* (where, x = 0.0, 0.1, 0.3) solutions (in CF with 5 mg mL^{-1}) and PEI solutions (in CF with 5 mg mL^{-1}) by targeted weight ratios, followed by stirring for 2 h at 150 rpm for the doping process. Then, 6 μL of the mixture solutions were drop-coated onto the alumina substrates which were patterned with Au parallel electrodes (25 and 70 μm of W and separation distance, respectively). Meanwhile, to demonstrate the flexible sensor platform, a flexible TPU substrate was prepared on which Cu/Au electrodes were deposited with 5 and 100 nm thickness, respectively, using an e-beam evaporation. Then, prepared IM-*x*/P-*y* sensing layers on PSS-coated glasses were floated onto the water by dissolving PSS sacrificial layer, followed by transferred onto TPU substrates.

Evaluation of Gas Sensing Performance

The sensing properties were measured using the homemade gas sensor testing equipment (Figure S6). After 2 h of stabilization in the N_2 atmosphere, the sensors were exposed to gas

analytes with controlled concentrations with a cyclic exposure of gas for 5 min followed by the introduction of baseline N_2 for 10 min. The resistance of the sensors was measured by using a 16-channel multiplexer (34902A, Agilent) at 4 s intervals. The resistance was converted into sensitivity values, *i.e.*, ΔR (= R_{gas} − R_b)/R_b (%), where R_{gas} and R_b denote resistance in target gas and baseline gas (N_2), respectively. The types of target gas were controlled to measure the selectivity tests and all the gases are N_2-balanced. The relative humidity level was controlled by using a humidity generator. The operating temperature was controlled by applying a DC voltage (DC power supply, E3647A, Agilent) to the microheater which is positioned on the backside of the alumina substrate.

Supporting Figures and Tables

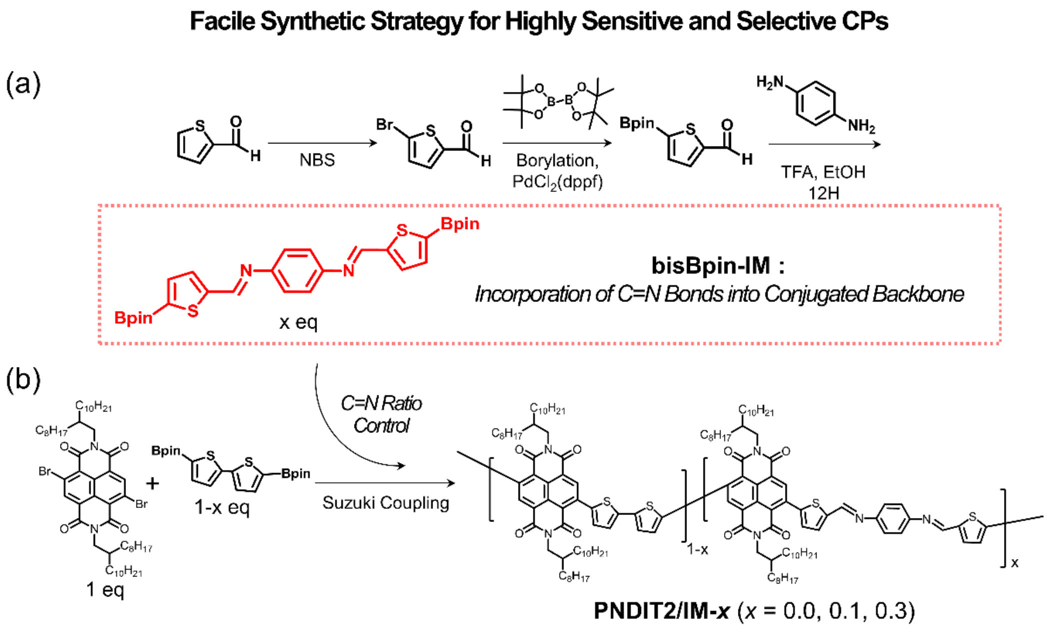

Figure S1. Schematic illustration of the synthesis of a) bisBpin-IM and b) PNDIT2/IM-*x*.

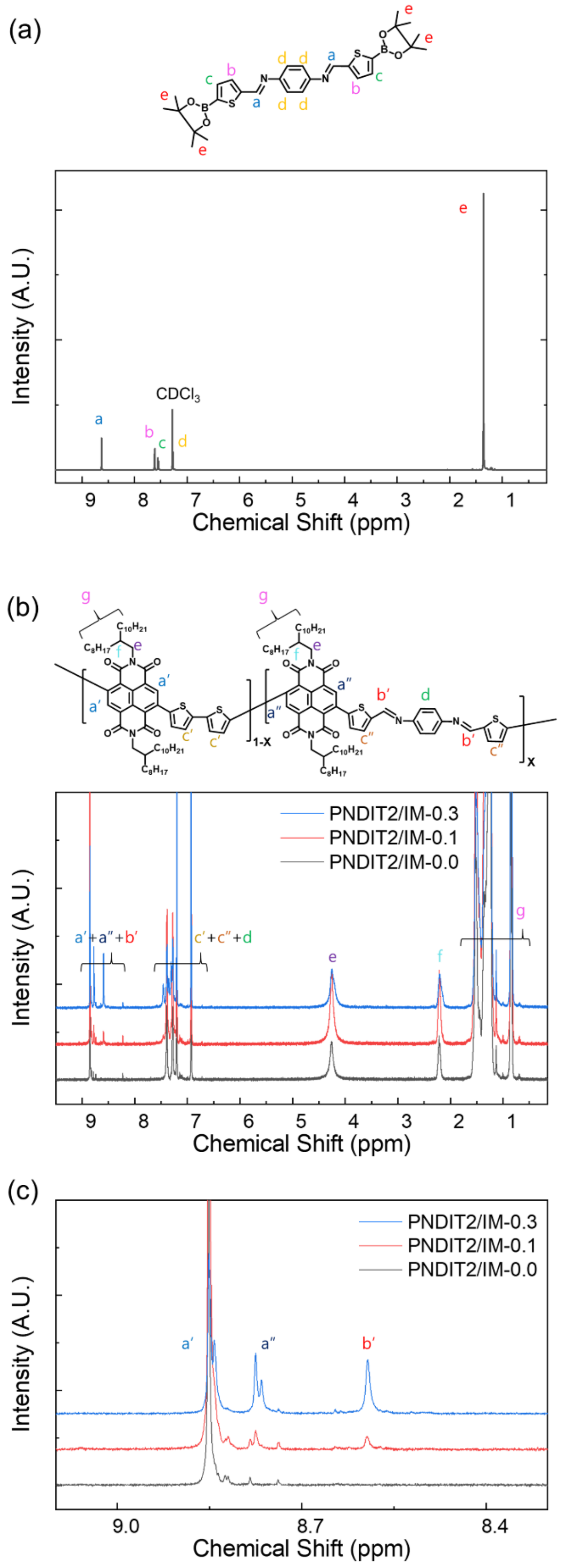

Figure S2. ^{1}H NMR spectra of a) bisBpin-IM and b) PNDIT2/IM-*x*. c) Magnified ^{1}H NMR spectra of PNDIT2/IM-*x* to determine the *x* values.

WILEY-VCH

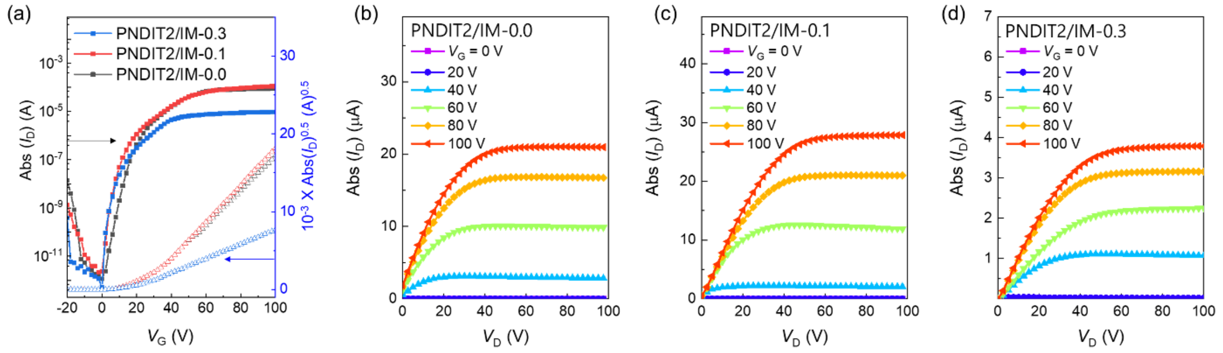

Figure S3. a) Transfer curves of OFETs fabricated with PNDIT2/IM-*x*. Output curves of OFETs fabricated with b) PNDIT2/IM-0.0 c) PNDIT2/IM-0.1, and d) PNDIT2/IM-0.3. (V_G = gate voltage, V_D = drain voltage, and I_D = drain current)

Table S1. Characteristics of PNDIT2/IM-*x* used in this study.

Polymers	M_n [a)] [kg mol^{-1}]	$Đ$ [a)]	x [b)]	μ_e [c)] [10^{-1} cm^2 V^{-1} s^{-1}]	I_{on}/I_{off} Ratio [c)]	V_{Th} [c)] [V]	COS [d)] [%]
PNDIT2/IM-0.0	50	1.9	0.0	2.3 ± 0.5	~ 10^6	16.5 ± 6.5	13 ± 2
PNDIT2/IM-0.1	65	2.1	0.1	2.7 ± 0.8	~ 10^6	25.5 ± 3.3	22 ± 3
PNDIT2/IM-0.3	57	2.4	0.3	0.4 ± 0.1	~ 10^6	20.2 ± 5.1	19 ± 2

[a)]Determined from the SEC traces calibrated with polystyrene standard; [b)]Determined from ^{1}H NMR spectra; [c)]Obtained from the OFET measurements; [d)]Calculated from the stress-strain graphs.

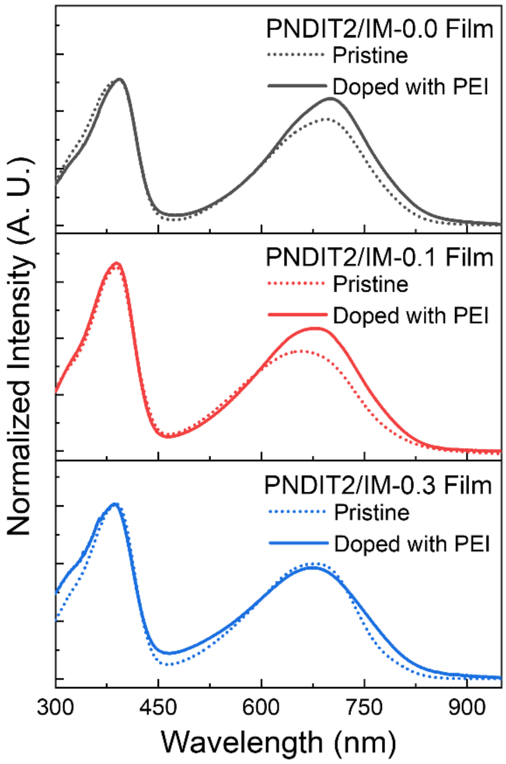

Figure S4. UV-vis absorption spectra of PNDIT2/IM-*x* films before and after doping.

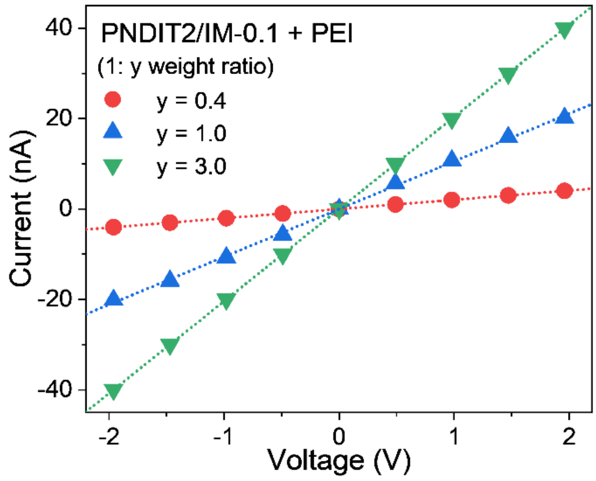

Figure S5. I-V curves of PNDIT2/IM-*x* films blending with different weight ratios of PEI.

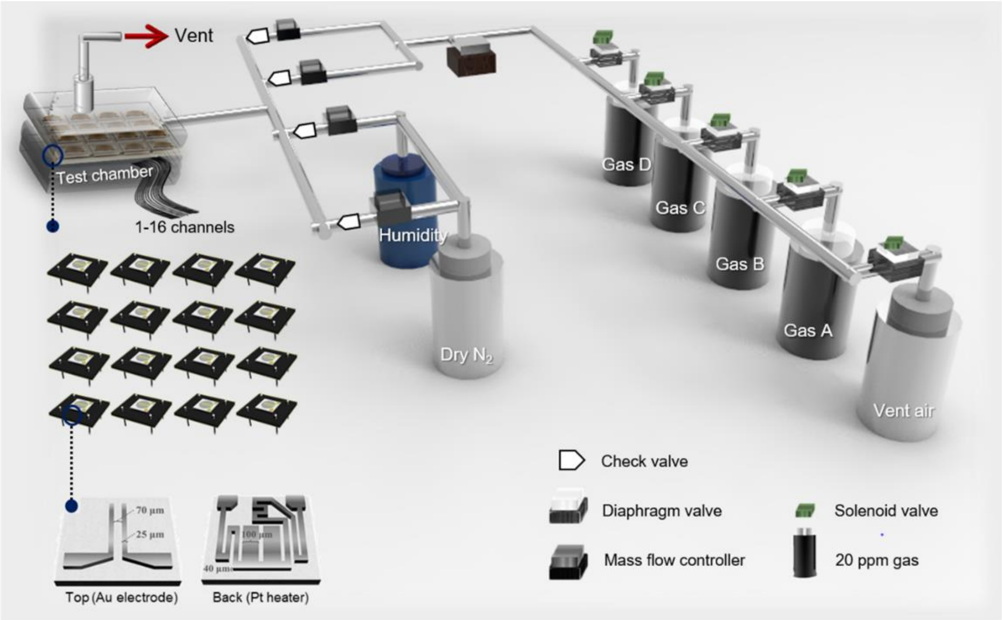

Figure S6. System for the gas sensor measurements.

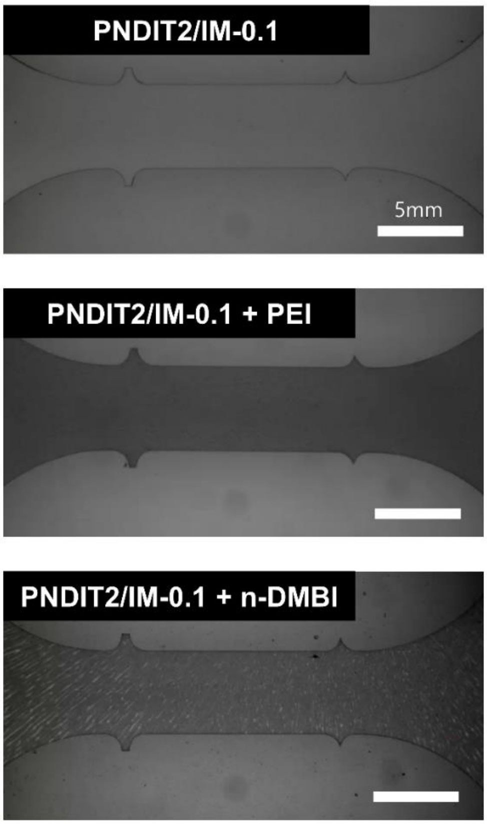

Figure S7. Optical microscopic images of PNDIT2/IM-*x* films under tensile test.

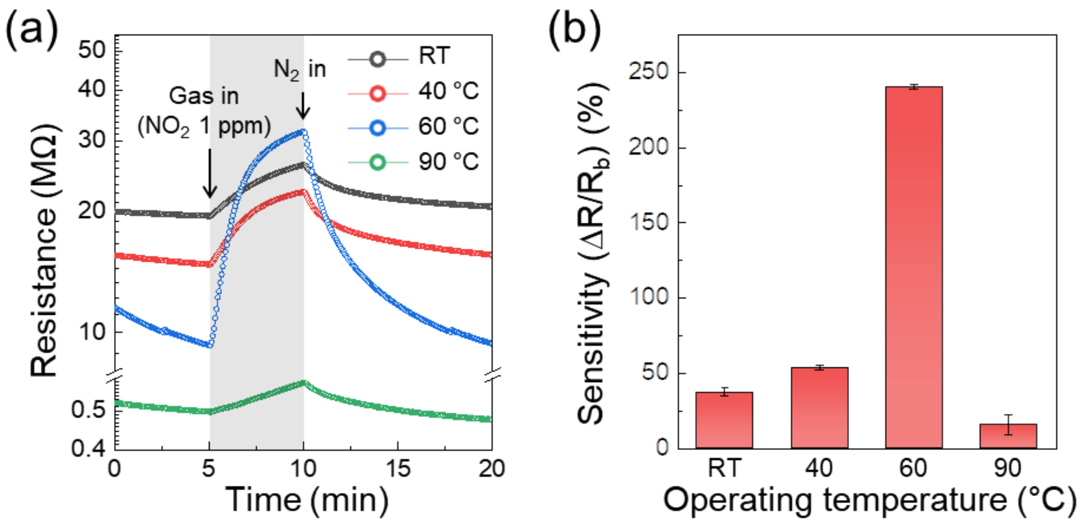

Figure S8. a) Dynamic resistance variations and b) sensitivity of IM-0.1/P-1.0 at controlled operating temperatures; RT, 40, 60, and 90 °C, respectively.

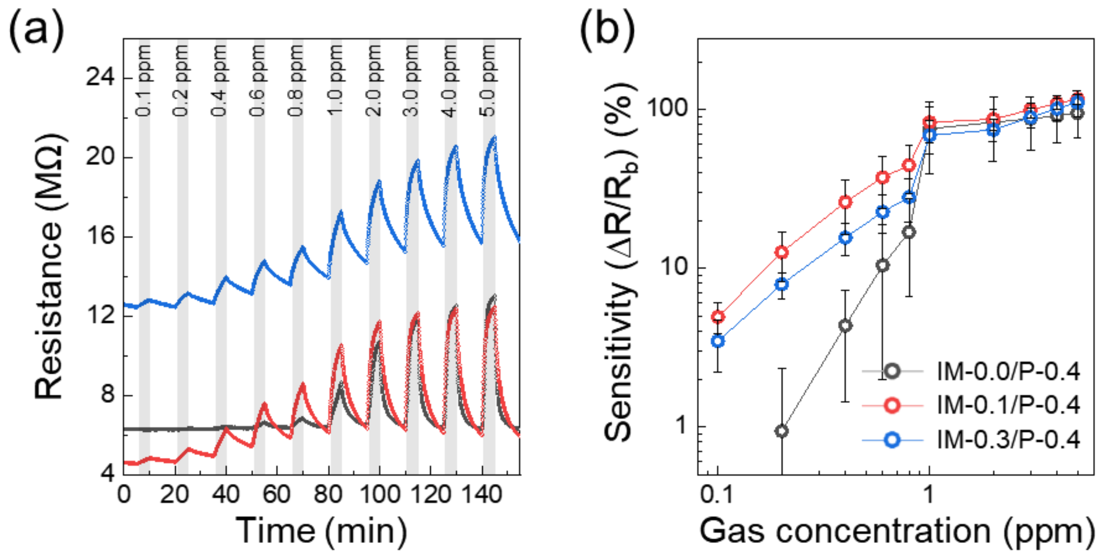

Figure S9. a) Dynamic resistance variations and b) sensitivity of IM-*x*/P-*y* with different IM contents (*e.g.*, *x* = 0.0, 0.1, and 0.3, respectively).

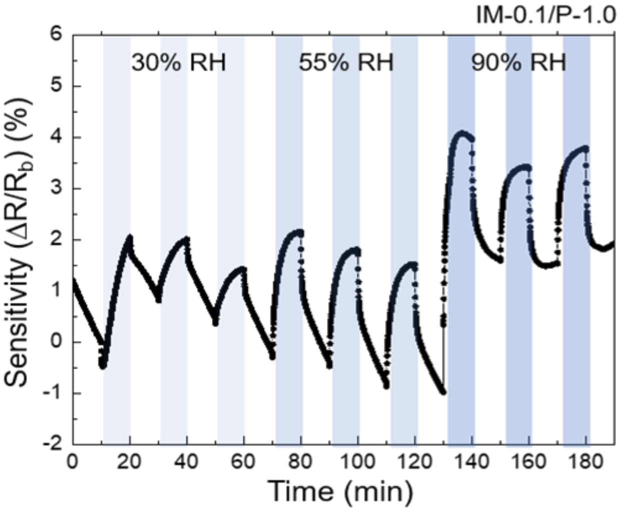

Figure S10. Humidity sensing properties of IM-0.1/P-1.0-based sensors upon exposure to 30, 55, and 90% RH in N_2 atmosphere.

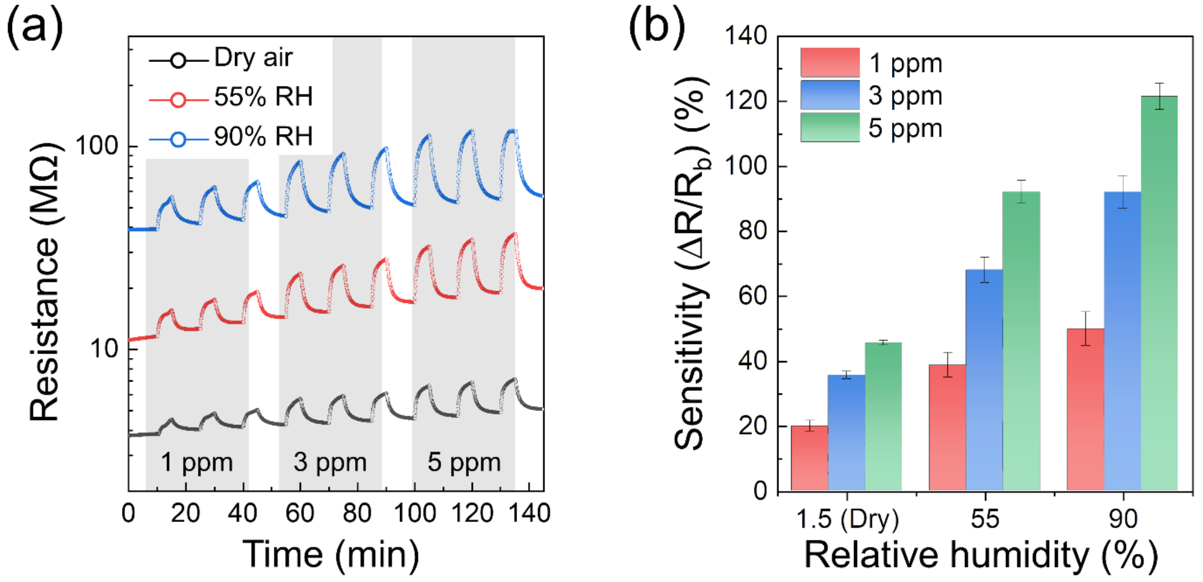

Figure S11. a) Dynamic resistance variations and b) sensitivity of IM-0.1/P-1.0 at controlled humidity conditions; 1.5 % RH (dry air), 55% RH, and 90% RH, respectively.

Table S2. Gas sensing properties of IM-0.1/P-*y*, where *y* = 0.2, 0.4, 1.0, and 3.0.

System	f_{PEI} [a)]	LOD [b)] [ppm]	$\Delta R/R_b$ [c)] [%] @ 1 ppm	Recovery time [d)] [sec] @ 1 ppm
IM-0.1/P-0.2	0.2	0.1	11.3	528
IM-0.1/P-0.4	0.4	0.1	83.0	400
IM-0.1/P-1.0	1.0	0.1	240.4	228
IM-0.1/P-3.0	3.0	0.1	93.8	96

[a)]Weight ratio of PEI and PNDIT2/IM-*x* in the sensor systems; [b)]LOD = limit of detection; [c)]Calculated resistance change variations, *i.e.*, sensitivity ($\Delta R/R_b$ [%]), by exposing 1 ppm of NO_2 at 60 ºC; [d)]Calculated recovery time to reach the 90 % of the original baseline resistance of the sensors during the recovery process (N_2 injection).

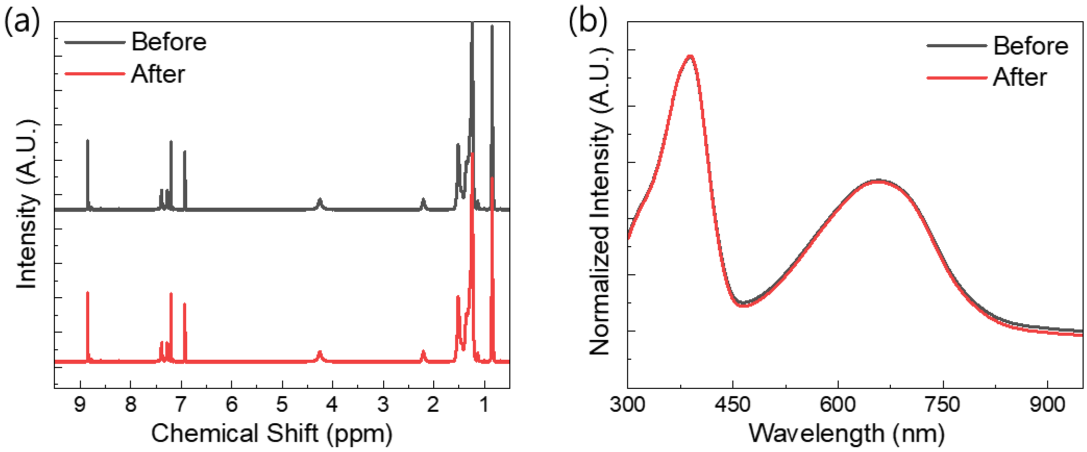

Figure S12. a) ^{1}H NMR spectra and b) UV-vis absorption spectra of PNDIT2/IM-0.1 before and after exposure NO_2 (1 ppm) for 3 days.

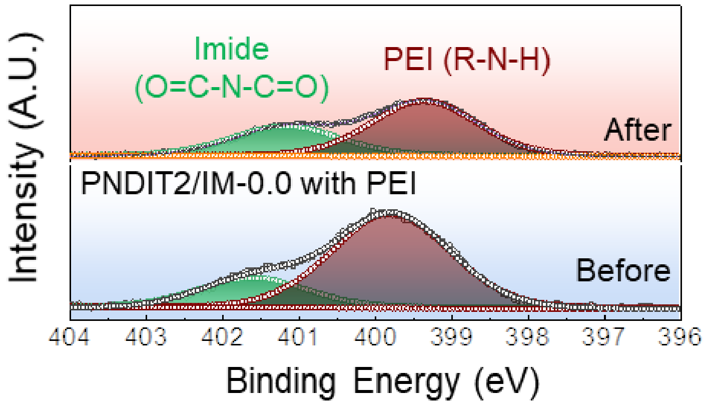

Figure S13. XPS analysis of N 1s spectra of the films of PNDIT2/IM-0.0 with PEI before (blue background) and after (red background) NO_2 exposure.
